# Fibrodysplasia Ossificans Progressiva: A Report of Four Cases

**DOI:** 10.7759/cureus.23392

**Published:** 2022-03-22

**Authors:** Mohamed Elamin, Ashraf Almutasim Ibrahim, Abdalla Omer

**Affiliations:** 1 Orthopaedics and Traumatology, Galway University Hospitals, Galway, IRL; 2 Orthopaedics and Traumatology, Omdurman Military Hospital, Khartoum, SDN

**Keywords:** acvr1/alk2, extraskeletal, great toe malformation, heterotopic ossification, fibrodysplasia, myositis ossificans progressiva

## Abstract

Fibrodysplasia ossificans progressiva (FOP) is a rare disease with less than a thousand confirmed cases. It is a severely disabling genetic condition that affects soft tissues and is characterized by progressive extraskeletal heterotopic ossification and great toe deformities.

The mode of FOP inheritance is autosomal dominant with no association to race, gender, or geographic distribution. While laboratory results and imaging studies support the identification of FOP, the diagnosis of this rare condition is mainly clinical. Recently, FOP has been linked to a mutation of the ACVR1/ALK2 gene that induces osteoblast activation.

We are reporting four cases of fibrodysplasia ossificans progressiva over a period of two years (2014-2016). Three out of four cases were treated conservatively. The first case was treated by excision of a bony bar, and the patient developed progressive bony formation and restriction of movement afterwards.

Almost always, FOP needs to be treated conservatively with non-steroidal anti-inflammatory drugs (NSAIDs) and gentle physiotherapy. Aside from anesthetic complications, surgical interventions provoke more bone formation, hence the recurrent joint restriction. Therefore, surgery should only be reserved for severely disabling deformities.

## Introduction

Fibrodysplasia ossificans progressiva (FOP) is a rare and severely disabling genetic condition that affects soft tissues. There are fewer than a thousand cases of FOP, with an estimated number of 3000 to 4000 affected patients [1,2]. FOP has autosomal dominant inheritance and has no racial, gender, or geographic distribution [1-3].

FOP is caused by a mutation of a bone morphogenetic protein (BMP) type I receptor gene named ACVR1/ALK2 [4,5]. Following mutation, this ACVR1 gene receptor induces osteoblast activation [5]. During the first few years of life, the child with FOP develops painful soft tissue swellings which are initiated by soft tissue injury, intramuscular injections, viral infection, or falls [2-6]. It is hypothesised that these injuries induce differentiation of mesenchymal stem cells through heterotopic ossification, which involves skeletal muscles, tendons, ligaments, and aponeuroses. Other theories propose that the accumulation of chondrocytes in extraskeletal muscles stimulates an inflammatory process following minor injury [6].

Pathognomonic features of FOP include congenital malformations of the great toes and progressive extraskeletal heterotopic ossification that forms normal bone in specific areas of the body, therefore restricting movement [3-5]. Children with FOP mostly have a normal appearance at birth except for malformations of the great toes such as short, deviated, and monophalangic toes [3-6].

The rate of progression of FOP widely varies with the heterotopic ossification found to first affect the neck, shoulder, upper back, and chest. FOP tends to progress in a centrifugal pattern from the axial skeleton to the extremities, with the lower extremity affected last. Of note, the diaphragm, cardiac and smooth muscles, tongue, and extra-ocular muscles are not affected by FOP [7].

Patients with FOP will mostly be wheel-chair bound by the third decade of life, and they usually have an average lifespan of 30-40 years [8,9]. Moreover, patients develop life-threatening conditions such as severe weight loss due to ankylosed temporomandibular joints as well as thoracic insufficiency syndrome. The latter was found to be the leading cause of death in most FOP patients [10].

The diagnosis of FOP is usually clinical. Laboratory investigations such as alkaline phosphatase support diagnosis. While imaging studies such as X-rays and computed tomography (CT) scans identify mature heterotopic ossification, other imaging modalities such as ultrasound, MRI, and most recently, positron emission tomography (PET) scans detect early ossification. Identification of the ACVR1/ALK2 gene mutation confirms the diagnosis [11].

Supportive therapy remains the mainstay of treatment for FOP as there is no definitive management yet. This includes avoidance of triggering factors such as trivial trauma, gentle physiotherapy, and occupational therapy. A short course of steroid injections was found to be helpful during flare-ups; however, injections in general, including some childhood vaccines, have to be avoided if possible to prevent further inflammatory derives [12].

Currently, there are few ongoing in vitro trials at the molecular level to inhibit the heterotopic ossification of FOP. Surgical options to excise these heterotopic ossifications have to be avoided as surgery represents a triggering factor [12,13].

## Case presentation

Report of four cases

Four cases were diagnosed with fibrodysplasia ossificans progressiva within a period of two years (2014-2016) at Omdurman Military Hospital, Khartoum, Sudan.

Case 1

**Figure 1:**
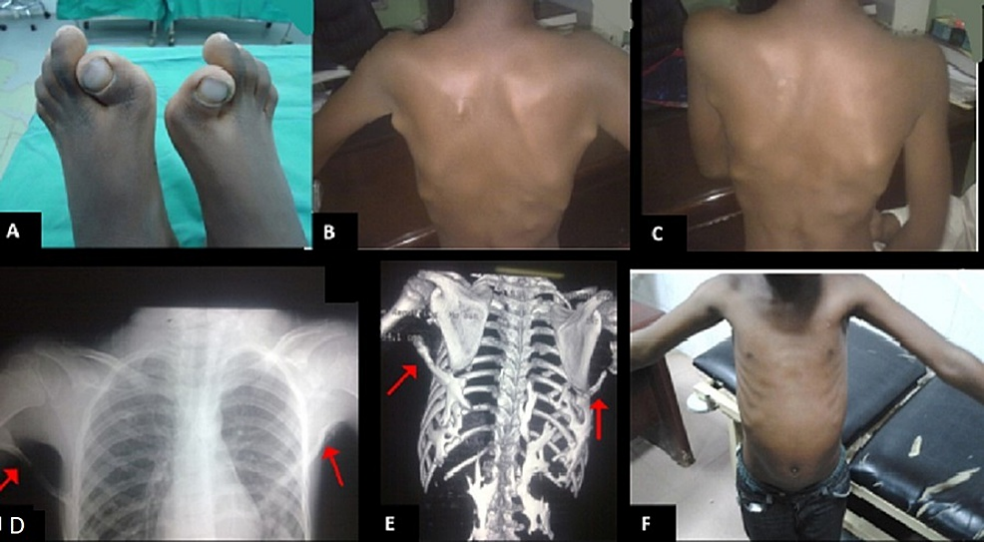
A 10 year boy with bilateral shoulder and back heterotopic ossification. (A) Big toe deformities; limited shoulder (B) abduction and (C) adduction; (D,E) chest X-ray and spine CT scan shows bony bridge (red arrows); (F) 4 months post-operative with gradual loss of shoulder range of movement.

A 10-year-old male was referred to our orthopaedic department for limited shoulder range of movement and truncal swelling noticed over a period of one year. He was the outcome of a normal delivery, passed through normal milestones, and completed his vaccinations. The patient and his parents denied a history of trauma or recent illness. His symptoms were progressive over one year, but there were no associated red flags or constitutional symptoms. There was no family history of a similar condition. On examination, the child was of average height and weight for his age and had no dysmorphic features. Back examination showed multiple non-tender solid lobules over the upper trunk region. These swellings were firmly attached to underlying structures but not to skin. Both the shoulder's active and passive ranges of the movement were limited to 15 to 45 degrees of abduction; stiff forward and extension movement. He had bilaterally deformed and short big toes (Figures 1A-1C). All laboratory investigations were normal. The diagnosis of FOP was made on clinical grounds. X-rays and CT scans were obtained to confirm the diagnosis (Figures 1D, 1E).

His parents were very keen on improving his shoulder abduction through surgery. Therefore, after explaining the benefits and risks of surgery (including infection, bleeding, nerve damage, and, most importantly, a recurrence of restricted movement), we got written informed consent from the parents. The patient had excision of a very superficial bilateral bony bar extending from the chest wall to both shoulders, which was limiting his shoulder abduction and adduction. He recovered well from surgery and could achieve 120 degrees of abduction. Unfortunately, he ended up with more restrictions on his movement just four months following surgical excision (Figure 1F).

Case 2

**Figure 2:**
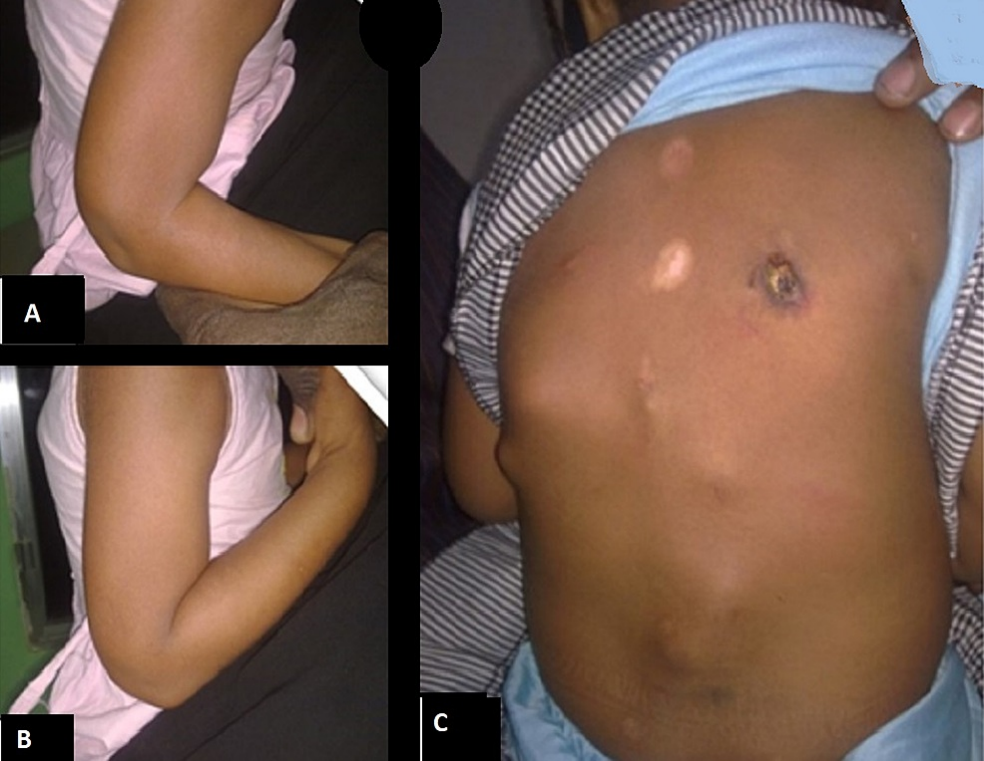
A five-year old girl with right elbow restricted active and passive (A) extension and (B) flexion. ROM (90-110 degrees). (C) Her back showing multiple bony lumps and traditional cupping therapy.

A five-year-old girl was referred by her paediatrician for increased stiffness of the trunk, shoulder and elbows six weeks following bronchiolitis. Her elbow active and passive range of motion was from 90 to 110 of flexion (Figure 2A-2B). Back examination showed multiple bony lumps similar to the first case, with traditional cupping therapy (Figure 2C). The child and her parents disappeared after suggestions to release her elbow flexors.

Case 3

**Figure 3:**
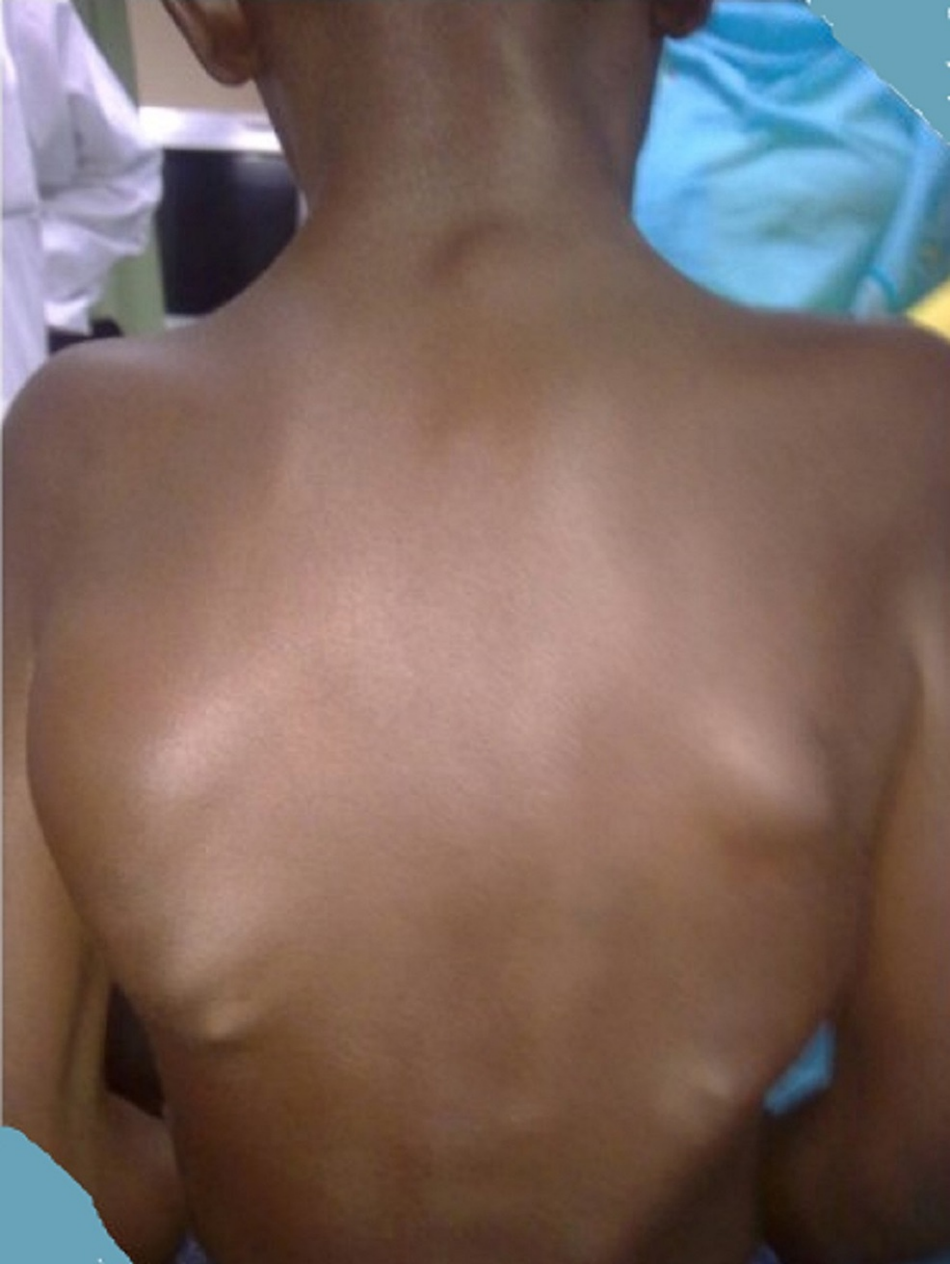
A 11 year boy with typical centrifugal pattern of fibrodysplasia ossificans progressiva at the neck, scapulae, and back.

An 11-year-old boy was brought in by his parents for restricted shoulder movement and a stiff neck. He showed the same bony lumps all over the back, neck, and front part of the shoulder (Figure 3). After reviewing imaging studies and laboratory results, he was sent to physiotherapy. 

Case 4

**Figure 4:**
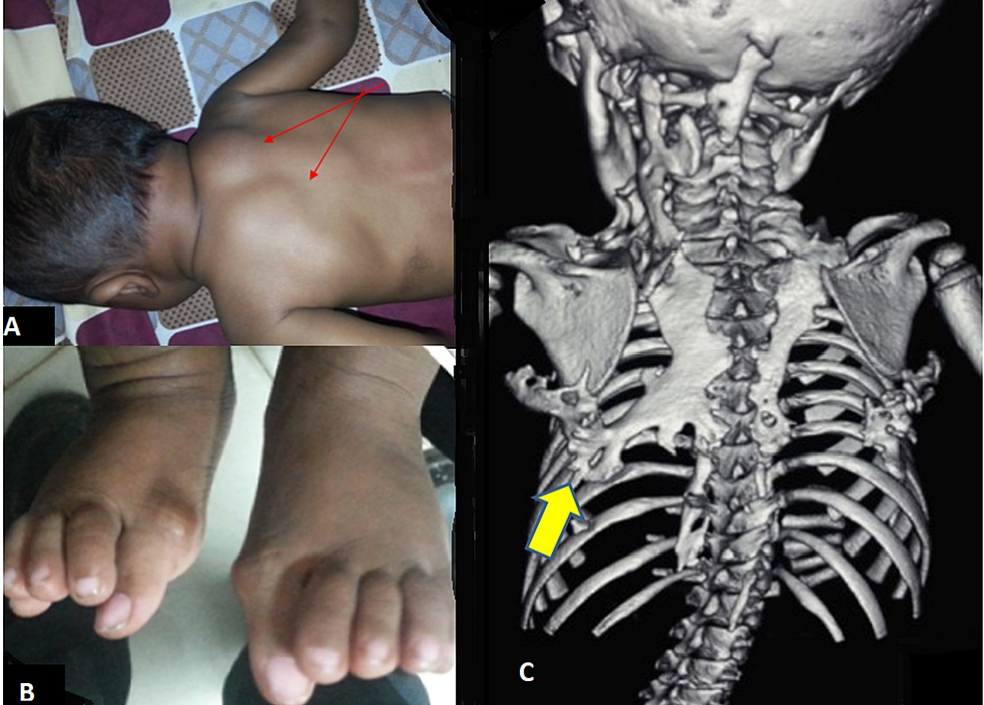
A seven months old female with pathognomonic clinical features of FOP: (A) heterotopic ossifications (red arrows) and (B) big toe deformity. (C) CT shows bony bridges (yellow arrow).

A seven-month-old female was referred by her paediatrician for delayed motor milestones and neck stiffness. She was the outcome of normal delivery with no dysmorphic features. Physical examination revealed the involvement of the trunk: the cervical region was most affected, as were both shoulders and the bilateral big toe deformity (Figure 4A-4B). The CT scan showed extensive bony bridges extending from the neck to the posterior chest wall and scapulae (Figure 4C). Her parents were counselled about the diagnosis and prognosis of this illness. This patient represented the youngest patient with the FOP in the world at the time of conducting this work.

## Discussion

Fibrodysplasia ossificans progressiva is a rare and severely disabling genetic condition that affects soft tissues. It is characterized by progressive extraskeletal heterotopic ossification and great toe deformities [1,2]. Diagnosis of this rare condition remains clinical [12,13]. All four cases we reported showed a characteristic pattern of heterotopic ossification on the neck, upper trunk, and upper extremities in addition to great toe malformation. Identification of the ACVR1/ALK2 gene, however, is required to differentiate FOP from other conditions such as osteosarcoma, lymphedema, soft tissue sarcoma, and desmoid tumours [1-14].

Currently, there is no satisfactory treatment for FOP; however, physicians have tried a short course of steroids within the first days of the flare-ups to halt the inflammatory process [8,15]. Moreover, prophylactic treatment includes preventing falls, respiratory illness, and viral infections. In addition, there are few in vitro molecular trials targeting the prevention of osteogenesis [15].

Of the four cases reported here, three were treated conservatively with non-steroidal anti-inflammatory drugs (NSAIDs) and physiotherapy. In the first reported case, the parents were aware of complications after surgery; however, they were insistently pressing towards the excision of the transverse bony bars bilaterally. Therefore, the orthopaedic team at Omdurman Military Hospital decided to proceed. The MDT meeting, including anaesthetics, orthopaedics, nurses, and physiotherapists, was held first to address the risks of intra-operative and post-operative complications that could follow intravenous/intramuscular injections, intubation, and jaw handling, as well as postoperative physiotherapy.

Unfortunately, the boy had a recurrence of shoulder stiffness within four months. Jayasundara et al. [16] showed a similar case of transient improvement of both shoulder and hip movement that was followed by progressive limitation of hip movement.

The orthopaedic team knew that they were going against most of the literature; however, there were few articles with short-term follow-up reporting successful excision. Ozkan et al. [17] and Moore et al. [18] reported satisfactory ROM following excision of heterotopic ossification in FOP patients.

Di Rocco et al. advised against surgical options because surgery represents a triggering factor, and they recommended a short course of oral steroids before and after surgery as a preventive measure if intervention is unavoidable [12]. Moreover, giving anaesthesia to patients with FOP is a difficult task due to the associated spinal column rigidity and ankylosed jaw [19].

## Conclusions

Fibrodysplasia ossificans progressiva is a rare genetic disabling condition that requires a high index of suspicion when examining a child with big toe deformity and heterotopic ossification. Laboratory results, including genetics and imaging studies, support the diagnosis of FOP, and treatment is almost always conservative.

Aside from the anaesthetic complications, surgical excision of heterotopic bone in FOP patients produces more bone formation, hence the recurrent restriction of joint movement. Therefore, based on our experience, we recommend that surgery should only be reserved for severely disabling deformities.
